# Predictive value of ACEF II score in patients with multi-vessel coronary artery disease undergoing one-stop hybrid coronary revascularization

**DOI:** 10.1186/s12872-021-02299-6

**Published:** 2021-10-10

**Authors:** Yanyan Li, Chuang Li, Dejing Feng, Qian Zhang, Kuibao Li, Yu Liu, Xinchun Yang, Lefeng Wang

**Affiliations:** Heart Center and Beijing Key Laboratory of Hypertension, Beijing Chaoyang Hospital, Capital Medical University, No. 8 Gongti South Road, Beijing, 100020 China

**Keywords:** ACEF II score, One-stop hybrid coronary revascularization, Major adverse cardiac and cerebrovascular events, Multi-vessel coronary artery disease

## Abstract

**Background:**

We aimed to investigate the predictive value of recently updated ACEF II score on major adverse cardiac and cerebrovascular events (MACCE) in patients with multi-vessel coronary artery disease (MVCAD) undergoing one-stop hybrid coronary revascularization (HCR).

**Methods:**

Patients with MVCAD undergoing one-stop HCR were retrospectively recruited from March 2018 to September 2020. Several prediction risk models, including ACEF II score, were calculated for each patient. Kaplan-Meier curve was used to evaluate freedom from cardiac death and MACCE survival rates. Differences of prediction performance among risk scores for predicting MACCE were compared by receiver operating characteristic (ROC) curve.

**Results:**

According to the ACEF II score, a total of 120 patients undergoing one-stop HCR were assigned to low-score group (80 cases) and high-score group (40 cases). During the median follow-up time of 18 months, the incidence of MACCE in the low-score group and high-score group were 8.8 % and 37.5 %, respectively (*p* *<* 0.001); and the cardiac death rate of the two were 2.5% and 12.5%, respectively (*p* < 0.05). Moreover, the cumulative freedom from cardiac death (97.5% vs. 86.8, *p* < 0.05) and MACCE (75.2% vs. 52.8%, *p* < 0.001) survival rates in the high-score group were significantly lower than in the low-score group. According to the Cox proportional hazards regression, the ACEF II score was an independent prognostic indicator for MACCE with hazards ratio (HR) 2.24, *p* = 0.003. The ROC curve analysis indicated that the areas under the curve (AUC) of MACCE from the ACEF II score was 0.740 (*p* < 0.001), while the AUC of MACCE from the SYNTAX score II CABG was 0.621 (*p* = 0.070) and the AUC from the EuroSCORE II was 0.703 (*p* < 0.001). Thus, the accurate predictive value of ACEF II score was similar to the EuroSCORE II but much higher than the SYNTAX score II CABG.

**Conclusions:**

The updated ACEF II score is a more convenient and validated prediction tool for MACCE in patients with MVCAD undergoing one-stop HCR comparing to other risk models.

## Background

Hybrid coronary revascularization (HCR), first proposed in 1996, is based on coronary artery bypass grafting (CABG) by grafting the left internal mammary artery (LIMA) to left anterior descending artery (LAD) while performing percutaneous coronary intervention (PCI) on non-LAD vessels [1]. By implementing minimal access, HCR actually lowers the incidence of peri-operative complications, such as infection and transfusion, which often complicate CABG [2, 3]. Compared to multi-vessel PCI-stenting, the employment of LIMA graft reduces the number of stents required, decreases the risk of stents restenosis and thrombosis, and further improves the long-term survival rate [4, 5]. However, in a prospective randomized trial, there were no significant difference between HCR and CABG in terms of the 1-year and 5-year rates of myocardial infarction, repeat revascularization, stroke or death [6, 7]. And the incidence of mid-term major adverse cardiac and cerebrovascular events (MACCE) remained up to 20–25 % for patients after HCR [8, 9]. Thus, early and accurate identification of MVCAD patients who undergo HCR at high risk of MACCE is critical.

Currently, several risk models have been established and in use for predicting adverse events after PCI and CABG, such as EuroSCORE and SYNTAX score. The age, creatinine and ejection fraction (ACEF) score is a convenient and effective risk model for predicting in-hospital mortality in patients undergoing elective cardiac surgery, with an accurate predictive power comparable to that of EuroSCORE [10, 11]. Subsequently, two variables including emergency surgery and pre-operative anemia were added into ACEF risk model, resulting in the updated ACEF II score. The ACEF II score has proven to be superior to the original model in discriminating and calibrating adverse outcomes in a large external area of cardiac surgery [12]. Though risk models could play a critical role in identifying high-risk patients following HCR, there is few evidence of which models are good predictors of poor outcomes. Hence, the objective of this study was to identify the best predictor for MACCE in patients with MVCAD referring for simultaneous HCR among current established risk models.

## Methods

### Patient population

A single-center and retrospective study was conducted at Beijing Chaoyang Hospital. Patients with angiographically confirmed MVCAD (stenosis > 50 % of the lumen diameter in at least two major coronary arteries that involving the LAD) who underwent one-stop HCR (CABG first then followed PCI in the hybrid operating room) were consecutively enrolled from March 2018 to September 2020. Exclusion criteria included: (1) underwent staged HCR but not one-stop HCR; (2) life expectancy less than 1 year due to severe concomitant non-cardiac diseases, such as malignant tumor or significant infection; (3) incomplete data for calculating ACEF II score; (4) contraindication to double antiplatelet therapy or drug-eluting stents; (5) lost during follow-up. Finally, 120 patients were enrolled into the analysis. The feasibility of one-stop HCR was at the discretion of the cardiac surgeons and interventionalists based on the results of coronary angiography and clinical characters at admission.

This study was approved by the institutional review board of Beijing Chaoyang Hospital and performed in accordance with the ethical standards laid down in the 1964 Declaration of Helsinki and its later amendments. All eligible patients had given written informed consent.

### Revascularization and pharmacological treatment

The approach for HCR stages, surgical grafting of the LIMA-LAD was performed without the assistance of cardiopulmonary bypass and through a ministernotomy access. After closure of the thorax, angiography of the LIMA-LAD graft by femoral artery was immediately performed to confirm the patency. Then PCI was performed for remaining lesions. For the antiplatelet schemes, Aspirin 100 mg/day was continued perioperatively, while clopidogrel was interrupted at least 7 days before the operation. Loading dose of clopidogrel 300 mg was given after confirmation of LIMA-LAD graft patency. And the patients received unfractionated heparin (70–100 IU/kg body weight) intravenously prior to stenting, to achieve an activated clotting time of > 250 s routinely. An early administration of aspirin 100 mg/day and clopidogrel 75/mg was performed on the first postoperative day. Then the dual antiplatelet therapy was maintained for 1 year and aspirin 100 mg/day was administered indefinitely.

### Data collection and risk scores calculation

The clinical and laboratory variables were collected from electronic medical records, such as age, sex, body mass index, left ventricular fraction (LVEF), haematocrit (HCT), white blood cell, brain natriuretic peptide and serum creatinine.

The ACEF II score was calculated according to Ranucci M et al. using the following formula: ACEF II score = Age/LVEF + 2.0 (if serum creatinine > 2.0 mg/dL) + 3.0 (if emergency surgery) + 0.2 × HCT points below 36 % [12], where the age was defined as completed years of the patients; the LVEF was defined as the percentage (%) at the closest pre-procedural assessment; serum creatinine (mg/dL) was the last recorded just before surgery. Due to the high technique difficulty and the requirement for close operators cooperation, it was uncommon to perform one-stop HCR in an emergency situation, thus urgent HCR was considered as a variable instead of emergency surgery (urgent surgery was defined as the EuroSCORE II). The assessment of pre-operative anaemia was based on the last HCT value before operation.

SYNTAX score I-II was based on the assessment of angiographic features by two professional interventionalists. Coronary artery disease is defined as a narrowing of more than 50 % of the lumen diameter in any major coronary arteries. SYNTAX score I-II was calculated on the basis of downloaded version from www.syntaxscore.com. For EuroSCORE I-II, pre-operative risk assessments were carried out for all patients by using the EuroSCORE systems. EuroSCORE I-II was evaluated according to the downloaded version from www.euroscore.pil-media.com.

### Clinical outcomes

The median follow-up time of the present study was 18 months after discharge. All patients were regularly reviewed via either telephone or outpatient interviews. Besides, hospital documents and outpatient clinic interviews for MACCE were collected as well. The study endpoints were the composite endpoints of MACCE, including cardiac death, re-hospitalization for myocardial infarction, repeated revascularization and stroke. And the composite endpoint was assessed by time to first event.

### Statistical analysis

SPSS (IBM, USA, version 25) and MedCalc (Seoul, Korea, version 19) were used for statistical analysis. Continuous variables were expressed as mean ± standard deviation (M ± SD) or median (interquartile range) in case of skewed distribution. Difference among groups was analyzed by Student’s t test or Mann-Whitney U test. Categorical variables were presented as percentages (%) and their statistical analysis were performed by the Chi-square test or Fisher’s exact test. Cox proportional hazards model analysis was used to determine the potential risk factors for MACCE and the results were presented as hazards ratio (HR) and 95 % confidential interval (CI). Variables with *p* < 0.1 were included into the multivariate model for further analysis, except EuroSCORE, EuroSCORE II, SYNTAX score II CABG and the variables incorporating into the ACEF II score. Discrimination performance of ACEF II score and other risk scores for MACCE was accessed by receiver operating characteristic (ROC) curve analysis, and their areas under the curve (AUC) were compared using a nonparametric approach. Kaplan-Meier curve with Log rank test was applied to detect difference in event-free survival rates between two groups. The ROC curve analysis was used to determine the optimal cutoff value of the ACEF II score (1.35, sensitivity: 68%, specificity: 75%). All statistical tests were two-sided and variables with *p* < 0.05 were considered statistically significant.

## Results

### Baseline characteristics

A total of 120 MVCAD patients who underwent one-stop HCR were recruited and divided into two groups according to the cutoff value of ACEF II score, 80 cases in low-score group (ACEF II score ≤ 1.35) and 40 cases in high-score group (ACEF II score > 1.35). The baseline demographic characteristics were presented in Table 1. Significant difference was observed among age, NYHA class, brain natriuretic peptide, urgent operation, transfusion, coronary intensive care unit-time, complete revascularization, SYNTAX score II CABG, EuroSCORE and EuroSCORE II between the two groups. Moreover, the incidence of diabetes mellitus in the low-score group and the high-score group were 35.0% and 57.5%, respectively, suggesting a significant difference between the two groups with *p* < 0.001. Further analysis also indicated the HCT and LVEF were significantly lower in the high-score group.

**Table 1:** Baseline demographics and clinical characteristics

Characteristics	Low-score group (n = 80)	High-score group (n = 40)	*p*-value
Age (years)	62.3 ± 9.8	69.1 ± 7.1	< 0.001
Male, n (%)	67 (83.8)	32 (80.0)	0.610
Body mass index (kg/m2)	26.6 ± 2.8	25.4 ± 4.1	0.065
*At admission*			
Hypertension, n (%)	56 (70.0)	31 (77.5)	0.386
Diabetes mellitus, n (%)	28 (35.0)	23 (57.5)	0.019
Hyperlipidemia, n (%)	53 (66.3)	33 (82.5)	0.063
Smoking, n (%)	49 (61.3)	18 (45.0)	0.091
Cerebrovascular disease, n (%)	16 (20.0)	10 (25.0)	0.554
PVD, n (%)	14 (17.5)	10 (25.0)	0.333
Previous PCI, n (%)	16 (20.0)	14 (35.0)	0.074
LVEF (%)	66 (62, 70)	55 (48, 63)	0.001
*NYHA class, n (%)*			
I-II	62 (77.5)	22 (55.0)	0.011
III-IV	18 (22.5)	18 (45.0)	–
*Clinical presentation, n (%)*			
Stable coronary artery disease	22 (27.5)	9 (22.5)	0.555
Unstable angina	46 (57.5)	19 (47.5)	0.300
NSTEMI	9 (11.3)	7 (17.5)	0.342
STEMI	3 (3.8)	5 (12.5)	0.115
*Culprit artery*			
LM, n (%)	30 (37.5)	16 (40.0)	0.564
LAD, n (%)	80 (100.0)	40 (100.0)	–
LCX, n (%)	50 (62.5)	30 (75.0)	0.357
RCA, n (%)	63 (78.8)	25 (62.5)	0.245
*Laboratory assessment*			
TC (mmol/L)	3.6 (3.1, 4.5)	3.7 (3.1, 4.1)	0.628
TG (mmol/L)	1.4 (1.1, 1.9)	1.2 (0.9, 1.7)	0.303
LDL-C (mmol/L)	2.2 (1.6, 2.7)	2.0 (1.6, 2.5)	0.215
HbA1c (%)	6.3 (5.8, 6.9)	6.2 (5.9, 7.7)	0.557
Serum creatinine (mg/dL)	0.79 (0.69, 0.92)	0.80 (0.72, 1.03)	0.207
BNP (pg/mL)	39.5 (24.0, 92.8)	92.5 (46.0, 294.8)	< 0.001
CK-MB (U/L)	1.00 (0.80, 1.63)	1.30 (0.60, 2.25)	0.231
TnI (ng/mL)	0.01 (0, 0.04)	0.02 (0, 0.71)	0.072
WBC (×10^9/L)	6.8 ± 1.6	7.5 ± 1.6	0.330
Neutrophil (×10^9/L)	4.2 ± 1.4	5.1 ± 1.4	0.145
Lymphocyte (×10^9/L)	2.4 ± 4.0	1.7 ± 0.51	0.263
HCT (%)	40.7 ± 3.1	34.7 ± 3.9	< 0.001
*Peri-operation*			
Urgent operation, n (%)	8 (11.2)	13 (32.5)	0.005
Elective operation, n (%)	71 (88.8)	27 (67.5)	–
Transfusion, n (%)	8 (10.0)	12 (30.0)	0.006
Reoperation, n (%)	2 (2.5)	3 (7.5)	0.196
Infection, n (%)	2 (2.5)	3 (7.5)	0.196
CCU-time (days)	4 (3, 6)	8 (3, 10)	0.025
Complete revascularization, n (%)	65 (81.3)	25 (62.5)	0.025
*Risk models*			
ACEF II score	0.96 ± 0.19	6.53 ± 2.55	< 0.001
SYNTAX Score	34.6 ± 6.7	33.9 ± 6.9	0.648
SYNTAX Score II CABG	25.4 ± 9.2	32.4 ± 9.0	< 0.001
EuroSCORE	7.1 ± 1.8	8.7 ± 1.8	< 0.001
EuroSCORE II	2.4 ± 1.6	4.9 ± 3.6	< 0.001

Low-score group, ACEF II score ≤ 1.35; High-score group, ACEF II score > 1.35

PVD, peripheral vascular disease; PCI, percutaneous coronary intervention; LVEF, left ventricular ejection fraction; NYHA, New York Heart Association; NSTEMI, non-ST-segment elevation myocardial infarction; STEMI, ST-segment elevation myocardial infarction; LM, left main artery; LAD, left anterior descending artery; LCX, left circumflex artery; RCA, right coronary artery; TC, total cholesterol; TG, triglyceride; LDL-C, low-density lipoprotein cholesterol; HbA1c, glycated hemoglobin; BNP, brain natriuretic peptide; CK-MB, creatine kinase isoenzymes; cTnI, cardiac troponin I; WBC, white blood cell; HCT, haematocrit; CCU, coronary intensive care unit

### MACCE characteristics between two groups

A total of 22 cases of MACCE (18.3 %) occurred during follow-up period. Compared to the occurrence of MACCE and cardiac death in the low-score group, with 8.8 % and 2.5 %, respectively, they were significant higher in the high-score group, with 37.5 % and 12.5 %, respectively (Table 2). While no significant difference was observed between the two groups regrading to the re-hospitalization for myocardial infarction, revascularization and stroke, their frequencies were higher in the high-score group.

**Table 2:** MACCE characteristics between two groups

Variables	Total (n = 120)	Low-score group (n = 80)	High-score group (n = 40)	*p*-value
MACCE, n (%)	22 (18.3)	7 (8.8)	15 (37.5)	< 0.001
Cardiac death, n (%)	7 (5.8)	2 (2.5)	5 (12.5)	0.040
Re-hospitalization for MI, n (%)	5 (4.2)	2 (2.5)	3 (7.5)	0.332
Revascularization, n (%)	6 (5.0)	2 (2.5)	4 (10)	0.094
Stroke, n (%)	4 (3.3)	1 (1.3)	3 (7.5)	0.107

Low-score group, ACEF II score ≤ 1.35; High-score group, ACEF II score > 1.35

MACCE, major adverse cardiac and cerebrovascular events; MI, myocardial infarction

### Freedom from MACCE and cardiac death survival rates between two groups

**Fig. 1:**
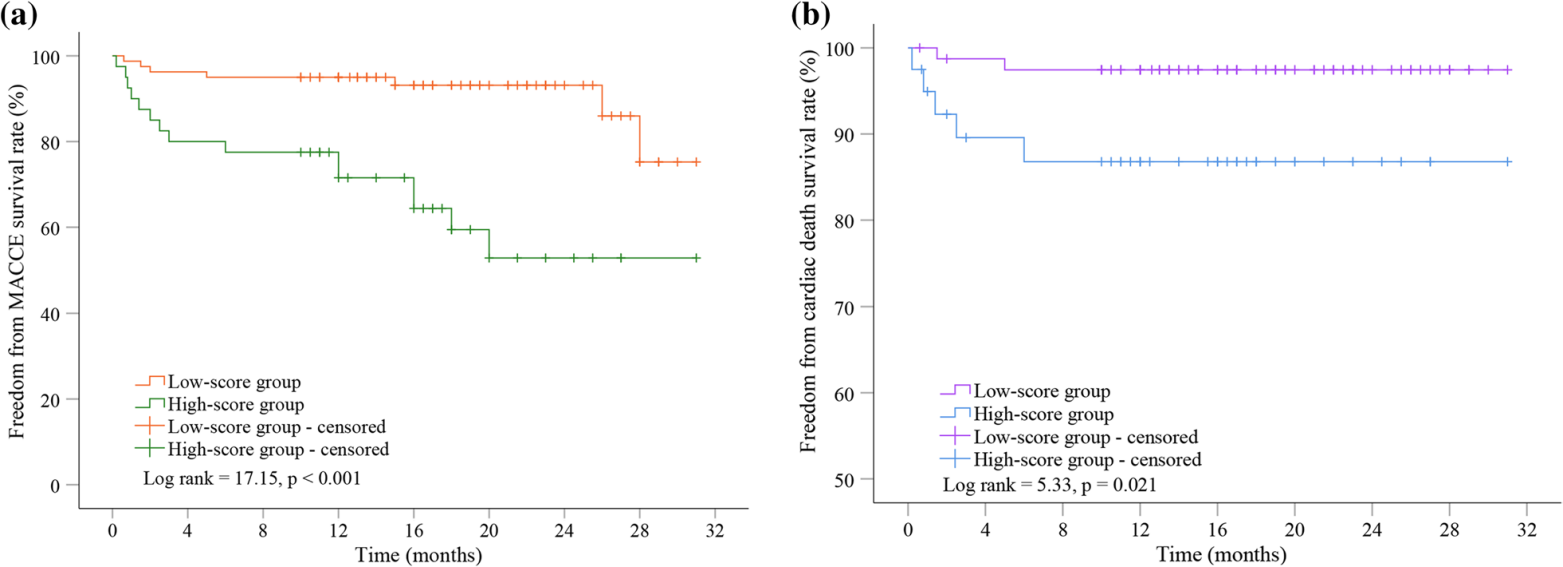
The Kaplan-Meier curve for cumulative freedom from MACCE (**a**) and cardiac death (**b**) survival rates between the low-score group and the high-score group. Patients in the high ACEF II score group were prone to suffer MACCE and cardiac death. MACCE, major adverse cardiac and cerebrovascular events

The Kaplan-Meier curve indicated that the cumulative freedom from MACCE survival rate was significantly lower in the high-score group than in the low-score group (75.2% vs. 52.8%, Log rank = 17.15, *p* < 0.001) (Fig. 1a). And patients in the high-score group had lower freedom from cardiac death survival rate than those in the low-score group (97.5% vs. 86.8%, Log rank = 5.33, *p* *=* 0.021) (Fig. 1b). In addition, about 50% of patients suffered MACCE within 3-month after HCR.

### Cox proportional hazards model analysis of risk factors for MACCE

The univariate Cox proportional hazards model analysis indicated that elevated ACEF II score was correlated with increased risk of MACCE (HR 2.60, 95% CI 2.41–2.77, *p* *<* 0.001), as well as diabetes mellitus, peripheral vascular disease (PVD), lymphocyte, HCT, EuroSCORE and EuroSCORE II (*p* < 0.05), as shown in Table 3. After multivariate adjustment, ACEF II score (HR 2.24, 95% CI 2.08–2.42, *p* = 0.003) and diabetes mellitus (HR 3.23, 95% CI 1.27–8.24, *p* = 0.028) remained to be independent predictors for 18-month MACCE of HCR patients.

**Table 3:** Univariate and multivariate analysis of MACCE

Variable	Univariate analysis		Multivariate analysis	
	HR (95% CI)	*p*-value	HR (95% CI)	*p*-value
Male	1.33 (0.44–3.98)	0.612		
Age	1.03 (0.99–1.08)	0.159		
Body mass index	0.90 (0.79–1.03)	0.114		
Hypertention	1.29 (0.48–3.51)	0.611		
Diabetes mellitus	3.18 (1.29–7.81)	0.012	3.23 (1.27–8.24)	0.028
Hyperlipdemia	1.15 (0.45–2.97)	0.770		
Smoking	0.89 (0.38–2.06)	0.777		
Cerebrovascular disease	1.85 (0.75–4.58)	0.181		
PVD	2.58 (1.03–6.47)	0.044	2.47 (0.89–6.89)	0.083
Previous PCI	1.02 (0.37–2.76)	0.973		
LVEF	0.97 (0.93–1.01)	0.138		
NYHA class	1.37 (0.57–3.32)	0.485		
Serum creatinine	1.00 (0.99–1.02)	0.737		
BNP	1.00 (0.99–1.01)	0.204		
CK-MB	0.93 (0.77–1.12)	0.439		
cTnI	0.99 (0.97–1.02)	0.745		
WBC	0.86 (0.65–1.13)	0.273		
Neutrophil	1.02 (0.75–1.38)	0.914		
Lymphocyte	0.30 (0.13–0.70)	0.006	0.67 (0.24–1.85)	0.435
HCT	0.85 (0.77–0.94)	0.002		
Transfusion	1.56 (0.57–4.22)	0.385		
Urgent operation	1.72 (0.23–12.96)	0.600		
CCU-time	1.02 (1.00–1.05)	0.081	1.02 (0.97–1.06)	0.474
Complete revascularization	0.95 (0.37–2.44)	0.916		
ACEF II score	2.60 (2.41–2.77)	< 0.001	2.24 (2.08–2.42)	0.003
EuroSCORE	1.48 (1.19–1.84)	< 0.001		
EuroSCORE II	1.27 (1.13–1.44)	< 0.001		
SYNTAX score	0.97 (0.92–1.02)	0.291		
SYNTAX score II CABG	1.05 (1.00–1.09)	0.055		

MACCE, major adverse cardiac and cerebrovascular events; PVD, peripheral vascular disease; PCI, percutaneous coronary intervention; LVEF, left ventricular ejection fraction; NYHA, New York Heart Association; BNP, brain natriuretic peptide; CK-MB, creatine kinase isoenzyme; cTnI, cardiac troponin I; WBC, white blood cell; HCT, haematocrit; CCU, coronary intensive care unit

### Predictive values of the ACEF II score versus other risk scores for MACCE

**Fig. 2:**
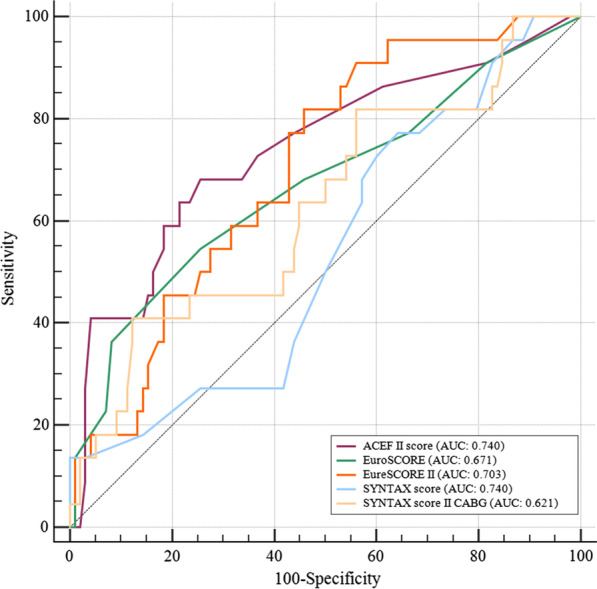
Receiver operating characteristic (ROC) curve analysis of ACEF II score compared with other risk scores in predicting MACCE. AUC, areas under the curve

At ROC curve analysis, ACEF II score (AUC: 0.740, *p* < 0.001), EuroSCORE (AUC: 0.671, *p* = 0.014) and EuroSCORE II (AUC: 0.703, *p* < 0.001) presented similar excellent discrimination in predicting MACCE (as shown in Fig. 2). Meanwhile, SYNTAX score (AUC: 0.536, *p* = 0.590) and SYNTAX score II CABG (AUC: 0.621, *p* = 0.070) had moderate discrimination in predicting MACCE. In addition, the ACEF II score had a sensitivity of 68.2% and specificity of 74.5 % for predicting MACCE. When comparing ROC curves, the ACEF II score was a more accurate predictor than both EuroSCORE [∆ AUC: 0.069, *p* = 0.333] and EuroSCORE II [∆ AUC: 0.037, *p* = 0.555], although no statistical significance. Both the SYNTAX score [∆ AUC: 0.204, *p* = 0.013] and SYNTAX score II CABG [∆ AUC: 0.119, *p* = 0.042] were significantly lower than the ACEF II score in predicting MACCE (Table 4).

**Table 4:** Predictive value of ACEF II score versus other risk scores for MACCE

	Single AUC analysis			Difference between AUC				
	AUC	95 %CI^b^	*p*-value	∆ AUC	95% CI^b^	SE^a^	Z statistic	*p*-value
ACEF II score	0.740	0.652–0.816	< 0.001	Reference	…	…	…	…
EuroSCORE	0.671	0.579–0.754	0.014	0.069	(− 0.071)–0.029	0.071	0.967	0.333
EuroSCORE II	0.703	0.613–0.783	< 0.001	0.037	(− 0.085)–0.519	0.062	0.591	0.555
SYNTAX score	0.536	0.442–0.627	0.590	0.204	0.043–0.366	0.082	2.477	0.013
SYNTAX score II CABG	0.621	0.535–0.714	0.070	0.119	(− 0.031)–0.260	0.072	1.853	0.042

MACCE, major adverse cardiac and cerebrovascular events; AUC, areas under the curve

^a^Hanley and McNeil (1983)

^b^Binomial exact

## Discussion

In the present study, we found that: (1) some MVCAD patients receiving one-stop HCR were still exposed to the high risk of cardiac death and MACCE; (2) the ACEF II score, which incorporates five clinical variables, was an independent risk factor for worse prognosis and had some potential for identifying patients at high risk of MACCE who underwent one-stop HCR; (3) similar to EuroSCORE and EuroSCORE II, the ACEF II score presented excellent predictive power and was superior to both the SYNTAX score and SYNTAX score II CABG in predicting MACCE.

MVCAD is documented in 40–60% of coronary angiography patients and has a poorer prognosis compared with single-vessel disease [13]. The optimal extent of myocardial revascularization in patients with MVCAD is controversial and consensus is lacking. According to guideline recommendations, conventional CABG is considered as the standard technique for the management of MVCAD [14]. However, saphenous venous grafts to non-LAD targets are more inclined to progressive stenosis, with occlusion rates ranging from 6 to 30 % as early as one year [15, 16]. In contrast, the 12-month rates of stents restenosis and thrombosis after PCI are less than 5 %, especially after deployment of new-generation drug-eluting stents [17]. Leveraging the advantages of both surgical and percutaneous techniques, HCR is expected to become a third coronary revascularization strategy for patients with MVCAD [18]. Moreover, the introduction of one-stop hybrid operating suites provides the opportunity for sequential surgical and percutaneous procedures, which can reduce the hospital stays and costs, also improve patients satisfaction [19, 20].

Notably, the high mortality and MACCE risk in MVCAD patients remains avoidable regardless of the optimized revascularization strategy [4, 21, 22]. The incidence of death and MACCE within 24 months after HCR was 5% and 19.3%, respectively [23]. Consistent with prior research, we found cardiac death and MACCE rates after HCR were up to 5.8 % and 18.3 %, respectively. Perhaps part reason of high risk of MACCE observed in one-stop HCR patients was the great complexity of coronary artery lesions, such as diffusion, calcification, bifurcation and chronic total occlusion. In such cases, PCI-stenting may be unable to achieve complete revascularization of non-LAD lesions, which is associated with a greater risk of short-term myocardial infarction, repeated revascularization and death [8]. According to the results of this study, the incidence of MACCE was predominantly driven by cardiac death (7 cases) and repeated revascularization (6 cases). Also, about 50 % of patients developed MACCE within 3 months after HCR. On the other hand, the present study indicated that diabetes mellitus was also a robust predictor for adverse events following HCR. Patients with diabetes mellitus had more severe and diffuse atherosclerosis and aggressive pathological progression, resulting in the higher rates of coronary restenosis and new stenosis after coronary revascularization, which were strongly associated with the MACCE [20, 24]. Further, due to the diffuse nature of the atherosclerotic process, patients with MVCAD are often complicated with PVD. Previous studies have shown that PVD is an independent predictor of adverse outcomes and poorer survival after CABG and PCI [25, 26]. Although not statistically significant after multivariate adjustment analysis, there was still an association between PVD and poor prognosis. In conclusion, early risk stratification, management and therapy of MVCAD patients are essential before initiating HCR strategy.

The ACEF score, consisting of only a simple triple variable, has good predictive power for adverse events in cardiac surgery and PCI [10, 27]. By incorporated anemia and emergency surgery into the original model, Ranucci M et al. validated that the new ACEF II score provide superior discriminative power to the original score [12]. Though it was originally designed for cardiac surgery, ACEF II score also presented excellent predictive power in patients treated with primary PCI and in patients with aortic dissections undergoing interventional thoracic endovascular aortic repair surgery, demonstrating a significant correlation between elevated ACEF II score and increased risk of subsequent adverse events [28, 29]. One-stop HCR is a simultaneous combination of surgical and interventional treatments including LIMA grafting to LAD and stents placement in other coronary lesions to manage MVCAD. Besides, previous studies have validated anemia and emergency surgery as prognostic markers for patients undergoing PCI or surgery [30, 31]. Due to the similarity in surgical technique and management, we hypothesized that ACEF II score could be used as a reliable risk stratification model for one-stop HCR patients. As mentioned above, the present study demonstrated that ACEF II score has similar discriminatory power for predicting MACCE compared with EuroSCORE and EuroSCORE II [32]. Meanwhile, ACEF II score significantly outperformed both SYNTAX score and SYNTAX score II CABG in predicting MACCE [33]. Therefore, the ACEF II score is a relatively convenient and user-friendly model for pre-HCR risk stratification.

### Limitations

Some limitations should be taken into account. First, the relatively small number of patients and the fact that the study was conducted in a single-center means that the prognostic value of the ACEF II score needs to be further confirmed in a large scale multi-center study. Currently, one-stop HCR is relatively difficult to implement because of the technical difficulty associated with it and the high expertise required of the operators, which is the main reason for the small sample size involved. Second, since the patients with MVCAD who only underwent one-stop HCR were recruited, it was difficult to validate our findings in patients with staged HCR. Third, because of missed follow-up angiographic imaging data on coronary artery disease progression after HCR, we were unable to further assess its impact on the endpoint events in this study. Fourth, due to the limitation of medical costs, the functional testing (noninvasive/invasive) of coronary lesions was not routinely used for patients, which might lessen the accuracy of the coronary disease diagnosis inevitably. Finally, the follow-up period was relatively short.

## Conclusion

ACEF II score, a relatively simple model, had been proved to be an independent predictor of MACCE in patients undergoing one-stop HCR. The ACEF II score could be used as a convenient and effective tool to guide physicians and surgeons in classifying high-risk MVCAD patients before HCR, thus potentially facilitating better clinical decision-making and treatment management.

## Data Availability

The datasets generated and analyzed during the current study are not publicly available due to the restrictions by the Beijing Chaoyang Hospital who is the data owner. The authors used this dataset under an agreement with the Beijing Chaoyang Hospital for the current study. If someone needs to access the data used in the study on reasonable requests please contact the corresponding author Lefeng Wang.

## Abbreviations

ACEF: Age, creatinine, ejection fraction
MACCE: Major adverse cardiac and cerebrovascular events
MVCAD: Multi-vessel coronary artery disease
HCR: Hybrid coronary revascularization
ROC: Receiver operating characteristic
HR: Hazards ratio
AUC: Areas under the curve
SYNTAX: Synergy between percutaneous coronary intervention with taxus and cardiac surgery
EuroSCORE: European System for Cardiac Operative Risk Evaluation
CABG: Coronary artery bypass grafting
LIMA: Left internal mammary artery
LAD: Left anterior descending artery
PCI: Percutaneous coronary intervention
LVEF: Left ventricular ejection fraction
HCT: Haematocrit
CI: Confidential interval
NYHA: New York Heart Association
PVD: Peripheral vascular disease
NSTEMI: Non-ST-segment elevation myocardial infarction
STEMI: ST-segment elevation myocardial infarction
LM: Left main artery
LAD: Left anterior descending artery
LCX: Left circumflex artery
RCA: Right coronary artery
TC: Total cholesterol
TG: Triglyceride
LDL-C: Low-density lipoprotein cholesterol
HbA1c: Glycated hemoglobin
BNP: Brain natriuretic peptide
CK-MB: Creatine kinase isoenzyme
cTnI: Cardiac troponin I
WBC: White blood cell
CCU: Coronary intensive care unit
MI: Myocardial infarction
